# Older migrants’ experience of existential loneliness

**DOI:** 10.1177/0969733021994167

**Published:** 2021-04-30

**Authors:** Jonas Olofsson, Margareta Rämgård, Katarina Sjögren-Forss, Ann-Cathrine Bramhagen

**Affiliations:** Malmö University, Sweden

**Keywords:** Experience, existential loneliness, interviews, migrants, older adults

## Abstract

**Background:**

With rapidly ageing population worldwide, loneliness among older adults is becoming a global issue. Older migrants are considered being a vulnerable population and ethical issues are often raised in care for elderly. A deeper sense of loneliness, existential loneliness is one aspect of loneliness also described as the ultimate loneliness. Making oneself understood or expressing emotions, have shown to be particularly challenging for older migrants which could lead to experience of existential loneliness. Ageing and being a migrant are potential triggers for experiencing existential loneliness. There appears to be, however, little known about being a migrant experiencing existential loneliness in old age.

**Aim:**

This study explored older migrants’ experience of existential loneliness.

**Research design:**

Qualitative study.

**Participants and research context:**

Data were collected through interviews (n = 15) with older (>65) migrants’ in Swedish nursing homes or senior citizen centres. A thematic analysis was performed to analyse the data.

**Ethical considerations:**

The study was conducted in accordance with the principles of research ethics.

**Findings:**

The result was described in terms of three themes: (1) Choices made in life, (2) seeking reconciliation with life and (3) thoughts about death and dying in a foreign country.

**Discussion:**

Ethical reflection and knowledge about how older migrants’ life story can lead to experiencing existential loneliness, could be of use in care for older migrants’.

**Conclusion:**

This study indicates that the experience of existential loneliness derived from being a migrant is a long-term and significant process. Migration was a hope of creating a meaningful life, the experience of existential loneliness occurred as migrants sought reconciliation with life, reflected upon their past choices, and thought about death and dying in a foreign country.

## Introduction

With rapidly ageing population worldwide, loneliness among older adults is becoming a global issue.^1^ Deterioration in health and higher risks for loneliness^2^ often leads to negative impact on well-being.^3,4^ Experiences of impending death, illness and physical limitations due to an inevitable ageing process are as well as not being met at a deep human level, common reasons for existential loneliness.^5,6^ There is no clear definition of existential loneliness, but it can be understood as an immediate awareness of being fundamentally separated from other people and from the universe, primarily through experiencing oneself as mortal.^7^ Existential loneliness can also be described as a deeper sense of loneliness. In critical situations in which a previously envisioned future and one’s basic security come under threat existential well-being may be negatively impacted and feelings of meaninglessness.^8–10^ From an existential point of view, this awakening triggers a crisis reaction.^11^ Besides being described as the ultimate aloneness, existential loneliness can also be seen as a part of being human^12^ that cannot be avoided during the course of life.^13^ However, existential loneliness can also be a peaceful positive experience if one chooses freely^14^ between a social network and solitude^15,16^ and lead to personal growth.^17^

For many countries, international migration is contributing to a change in population age structures as migrants who remain in the country of residence, becomes part of indigenous older adults.^18^ Not being able to express oneself nor being understood^19^ might trigger the feelings of existential loneliness for older migrants and possibly affecting ones autonomy.^20,21^

The fundamental basis of being at home^22^ in the world is lacking when existing in-between different identities, that is, maintaining a cultural value while adapting to new forms of living.^23^

As described above, both ageing and being a migrant are potential triggers for existential loneliness. It is, however, unclear whether the experience of existential loneliness is different for older migrants or not. In care for older migrants, ethical challenges can be raised due to miscommunication and different cultural values.^18^ In an intercultural setting, it is of importance for health care professionals to provide ethical nursing care with intention to do good and not harm^24^ to vulnerable persons.^25^ This means creating possibilities for older migrants to participate in their own care even if the values and beliefs of health care professionals might differ from theirs, that is, cultural competence. It is of importance to be aware of one’s own cultural world view and value diversity.^26^ Being aware of one’s own cultural world view, value diversity and knowledge about older migrants’ experience of existential loneliness are prompted in care for older adults.

Existential loneliness has been considered in several healthcare contexts such as psychiatry, chronic illness, palliative care, and care for older adults.^27–29^ However, to best of our knowledge, there appears to be sparse knowledge regarding being a migrant experiencing existential loneliness in old age, which contributes with understanding of existential loneliness by older migrants. Therefore, the current study aims to explore older migrants’ experiences of existential loneliness.

## Method

A qualitative descriptive study was performed to understand the experience of existential loneliness among older migrants. Data were collected through interviews^30^ and analysed thematically using the method of Braun and Clarke.^31^

### Study setting

This study took place in a multi-cultural city in the southern part of Sweden during autumn 2018 and spring 2019. The city has one of the highest number of foreign-born inhabitants, approximately one third, represented by 184 different nationalities in Sweden compared with about 20% in Sweden in total.^32^ The study took place at either nursing homes or senior citizen centres (SCC). The care of older people in Sweden is a public responsibility, funded by municipal taxes and government grants. The municipality is responsible for care of older people, but it can be outsourced to public or private actors. Nursing homes provide round-the-clock care for older people due to multi-morbidity and, cognitive and physical impairments. Besides providing care for physical needs, nursing homes also care for emotional and social needs. Depending on the size of the nursing home, one or two registered nurses are on-call to guide the care during office time from Monday till Friday, or even on weekends in some cases. Staff nurses and healthcare assistants are the main providers of around-the-clock care in nursing homes. A primary-care physician, most often working at a healthcare centre close to the nursing home, cares for most of the residents. Nursing-home care is commonly provided until the resident’s death. SCCs, on the other hand, are centres for older persons to congregate located in several parts of the city. SCCs provide, among other things, fellowship, physical activities and musical entertainment to fulfil older people’s physical, emotional and social needs. SCCs are most often managed by elderly pedagogues, social workers, or assistant nurses.

### Sampling and recruitment

To attain variation in the age and experience of people in care for older adults, persons from nursing homes and SCCs were recruited. In the first step of the recruitment process, the first author (J.O.) contacted the administrative heads of nursing homes and SCCs to inform them about the study. In nursing homes, a nurse identified prospective participants and informed them about the study. If they expressed interest, the nurse contacted the first author, who informed the prospective participant about the study and then made an appointment for an interview.

Three SCCs’ were contacted. At the SCCs’, the first author (J.O.) contacted the manager and then visited the centres to inform prospective participants about the study and to answer questions before appointments were made. The inclusion criteria were adults aged 65 or older, born outside of Sweden and able to communicate in Swedish.

### Study participants

A total number of 15 older adults agreed to participate in the study. Seven of the participants were recruited from five nursing homes. Four of the nursing homes were managed by the municipal government and one was privately outsourced. Eight participants were recruited from three SCCs. The characteristics of participants are presented in Table 1.

**Table 1.** Characteristics of participants (n = 15).

Age	
* Range in years (median)*	*66–99 (82)*
Gender	
* Women*	*12*
* Men*	*3*
Country of origin	
* Austria*	*1*
* Germany*	*2*
* Hungary*	*1*
* Iran*	*1*
* Poland*	*3*
* Portugal*	*1*
* Serbia*	*5*
* Slovenia*	*1*
Years in Sweden	
* Range in years (median)*	27–73(50)*

*2 participants missing out.

### Data collection

Interviews were conducted between October 2018 and May 2019 by the first author (J.O.). The interviews took place in the participants’ nursing home, or SCC, except for one interview that was held in a café upon the participant’s request. The participants were asked open questions related to their experience of loneliness and existential loneliness such as ‘Have you experienced existential loneliness?’, ‘What was the reason for leaving your country of origin?’, ‘Is there anything you are missing and longing for from your country of origin?’ and ‘What do you think about the future?’ Existential loneliness was explained to the participants as a deeper sense of loneliness. One pilot interview was held to evaluate the validity of the questions prior conducting the study, however, no changes were made, and the pilot interview was included in the final analysis. The interview length ranged between 17 and 75 min. The interviews were transcribed verbatim by the first author. Three participants felt insecure about being tape recorded during the interview and specifically asked not to be recorded, and the first author therefore transcribed their answers upon consent.

### Data analysis

A qualitative descriptive approach to thematic analysis was used to find patterns within the data.^31^ Using inductive coding strategy, the themes were identified at a latent level to identify the underlying content in the data. The analysis was conducted in six steps. The first author (J.O.), at first, familiarized himself with the material by transcribing and then reading through all interviews. The interviews in this step were also read by the other authors (A.C.B., K.S.F. and M.R.). During the first step, initial thoughts concerning data were written down. The second step involved highlighting interesting text with a coloured pen and coding features across the data according to the aim. Thereafter, in the third step, codes were gathered into themes (Table 2). In the fourth step, all authors reviewed the themes and discussed them until they had achieved agreement before defining and renaming the themes in the fifth step. Thereafter, in the sixth step, the final report was written. The process for analysis was manually conducted.

**Table 2.** Extracts from data to exemplify the themes.

Data extract	Code	Theme
*Everything was destroyed by lack of health. That’s what I am telling you now. There is no value to money if you are not well. Health cannot be paid for. That is the way it is. // I cannot change anything. Now I can just accept and maybe live for a few more days. I don’t know (Participant 11)*	Acceptance of the story of life	Searching for reconciliation with life
*Yes, we want to meet with our own people. It is not easy. We want to meet with our own people. There are some from Poland here// You know, we are sensitive (Participant 5)*	Need to belong	
*Loneliness in togetherness, that is what it is. Very much. Our friends have either moved or are very old (Participant 15)*	Alone despite people around	
*When my son died, I wanted to move back [to country of origin] but I couldn’t. The cemetery is only a ten minutes walk away from where I live (Participant 1).*	Ambuigity	
*It is not easy to accept (your choices in life). //But it is best to accept (Participant 10)*	Acceptance	Choices made in life
*It would have been different and easier if we lived in Serbia. Family is there and it is easier to have contact with your neighbours. It is different in Sweden (Participant 3).*	Existential guilt	
*I know we will meet again on the other side. I have stayed on. I am not done with everything I have to do before I go. We will all walk that way. For sure. We, who stayed on, have to go on fighting with life (Participant 14)*	Hope about tomorrow	Thoughts about death and dying.
*Nu är sista tiden. Det är jag glad för eftersom livet har varit svårt. Och jag är inte rädd för sista tiden. Tiden går och jag glömmer med tiden också. Ibland är det bra att glömma (Participant 2)*	End of days	

### Ethical considerations

This study was performed in accordance with the ethical guidelines given by the Helsinki Declarations^33^ and approved by the Regional Ethical Review Authority in Lund, Sweden (2018/544). All participants were informed of the study requirements and the voluntary nature of participation and their right to withdraw at any point without needing to give any explanation for doing so. The participants were provided written consent prior to participation. In this paper, confidentiality was ensured via pseudonyms to protect participants’ anonymity. Confidentiality was repeated before starting the interview, and participants were told again how the data were stored. The study involved vulnerable older people and the questions asked could be perceived as emotional and stressful. The participants were also told that they did not have to answer all questions. The interviews were performed by the first author, who had previous experience in performing interviews with older and vulnerable people and who strove to be sensitive and respectful. During the interview, the first author remained aware of the participants’ reactions and ensured that they could stop the interview. If showing signs of agitation or despair, the participants were asked if further contact with the first author, nurse, or a caregiver was wanted. Data were stored securely and anonymously in compliance with the Data Protection Act.

## Findings

Migrants experience of existential loneliness are described in terms of three themes: *Choices made in life, Searching for reconciliation with life* and *Thoughts about death and dying in a foreign country.*

### Choices made in life

Existential loneliness was expressed when reflecting upon choices made in life. If expectations for leaving the country of origin were not met, the experience of existential loneliness occurred along with existential guilt towards their family and themselves. The participants then questioned whether their life would have been easier if they had not left their countries of origin. This existential guilt was more obvious for those who had moved freely of their own choice. Those who felt they had no choice but to leave their country of origin due to war or other difficult circumstances, did not express existential guilt to the same extent.

Another reason for experiencing existential loneliness was expressed by the participant when struggling to accept their life choices made past in life. Accepting choices was important for to incorporate earlier negative situations:

It is not easy to accept (your choices in life). //But it is best to accept. (*Ana 73*)

### Searching for reconciliation with life

The participants had left their countries of origin in the hope of creating a meaningful life. As life went on, difficult circumstances made them experience a sense of meaninglessness. In the ongoing process of searching for reconciliation with their life story, feelings of existential loneliness occurred.

In reflecting upon ageing in the country of residence rather than their country of origin, participants raised existential questions such as the meaning of life. Emigration was, for all participants, an act of major significance in their life. The loss of an anchor to their country of origin caused participants to experience existential loneliness, regardless of whether they emigrated due to difficult circumstances like war, or political instability or if emigration was chosen freely.

Participants felt vulnerable ageing in the country of residence, especially when they were dependent on others for their daily lives due to ageing, physical decline, or illness. Dealing with the loss of one’s health and one’s next of kin brought feelings of existential loneliness to mind:

Everything was destroyed by lack of health. That’s what I am telling you now.

There is no value to money if you are not well. Health cannot be paid for. That is the way it is. // I cannot change anything. Now I can just accept and maybe live for a few more days. I don’t know. (*Marija 83*)

There was also a sense of ambiguity expressed by participants due to the feeling of being between two cultures. As an example, parents and/or spouses may want to be cared for and buried in their country of origin, while the participants’ children, being second-generation migrants, do not share the same ambivalence.

The lack of family in the country of origin was a reason for experiencing existential loneliness. Therefore, participants expressed a need to belong. Even if it was not easy, several participants tried to connect with other migrants with similar experiences. Through relationships, an important sense of belonging was created by sharing an understanding of one’s culture and communication in their own language. One of the participants living in a nursing home stated:

Yes, we want to meet with our own people. It is not easy. We want to meet with our own people. There are some from Poland here// You know, we are sensitive. *(Khrystyna 99)*

Even if living in a relationship, feelings of existential loneliness could occur when participants were not able to share their life stories. Experience of existential loneliness also occurred as friends from their country of origin were no longer able to visit:

Loneliness in togetherness, that is what it is. Very much. Our friends have either moved or are very old. (*Sara 81*)

Although the participants expressed the importance of belonging, they also shared experiences of the feeling of not belonging. If they were able to visit their country of origin, a sense of being an outsider and no longer belonging occurred, leading to feelings of existential loneliness. Several participants also struggled to find their identity in a new country. Even though they spent most of their lives in Sweden, the participants stated that they would never identify them self as Swedish. This was expressed in various ways by the participants. For example, some participants, found that their education and status from the country of origin was of no use in their new country:

I was 17 years old when I got to Sweden//You are a foreigner both in Sweden and when I go back to Serbia. It feels like living in a limbo. (*Silvija 67*)

One major factor to belonging was knowing the language. Otherwise, a language barrier created a distance, leading to a feeling of being left out and isolated:

It is important to know the language//Otherwise you are completely left out, like a zombie. You feel trapped. (*Ania 68*)

### Thoughts on death and dying in a foreign country

EL was exposed to the utmost by participants’ awareness of their coming deaths and the fact that their remaining lifetimes may be measured in days. Participants who were close to death and who needed care in their daily lives due to illness, grief, or loneliness struggled to cope with feelings of existential loneliness induced by their suffering. Thoughts on death and dying brought about a longing for participants’ countries of origin and their familiar cultures. Music, food, and relationships became more important. When talking about the future, thoughts about where their life would end and where they would be buried were expressed. Not being able to be buried next to family members in their country of origin created feelings of existential loneliness. In the valley of death, thoughts formerly forbidden were now spoken aloud. Death was seen both as a welcoming deliverer for the older migrants, as well as something frighteningly inevitable:

I have no wish to live anymore. I pray every night. Dear God. Let me fall asleep and not wake up anymore. I do not want to wake up. I mean it. Now I do not want to fight anymore. I am done. I do not hear; I am nearly blind; I can no longer walk. Why the hell should I fight? I have fought. I have fought for life//I have been crying many nights. I will never see my country of origin again. That hurts so. I can no longer visit my mother’s grave. (*Fritz 92*)

For participants close to the end of days, it was hard to let go. There was still a hope for a better tomorrow. This hope was especially addressed to the family they were leaving behind. Existential loneliness was connected to the uncertainty not knowing what will happen after death. Despite this uncertainty, there was a hope of reuniting with loved ones. The positive expectations for good circumstances in the future, were connected to reuniting with family they were no longer able to meet, both from their current country and their country of origin, regardless of whether death was near or if there were expectations for longer life. For the participants that were able to see their life in a wider context, the hope for a positive future gave the participants energy to go on and to cope with existential loneliness:

I know we will meet again on the other side. I have stayed on. I am not done with everything I have to do before I go. We will all walk that way. For sure. We, who stayed on, have to go on fighting with life. (*Lidija 73*)

## Discussion

The aim of this study was to explore older migrants’ experience of existential loneliness. Our findings show that the long-term process of migration is an aspect of migrants’ experience of existential loneliness in old age as they in perpetually (1) accept choices made in life, (2) search for reconciliation with life and (3) cope with thoughts about death and dying in a foreign country. These can be seen as existential loneliness triggers for older migrants, despite ageing, losses, and being close to death. The expression of existential loneliness is expressed depends upon the individual.

The participants had lived outside of their country of origin for most of their lives and most were well-integrated in Sweden. However, our findings showed that there are triggers for experiencing existential loneliness linked to being a migrant. Whether enforced or voluntary, migration was a significant act for all participants. Migration was also stated to be a long-term process in which the individuals experienced themselves as alone, bringing existential aspects to the fore. It is known that people in old age spend time reflecting upon their lives^34,35^ in the need to find life meaningful. The concept of meaning is crucial for human beings. Furthermore, the search for meaning in life is a basic human need, and the human being has the ability to find meaning in difficult circumstances.^36^ More so, the human being is condemned to meaning.^37^

Several participants in this study emigrated due to difficult situations in their country of origin, in the hopes of creating a meaningful life for themselves and their families. Yet in old age, the migrants in our study questioned whether they made the right choice. Every human being makes choices in life,^38^ and also carries the responsibility for choices made in life. These choices often bring existential issues to the fore,^39^ something agreed upon by the older migrants in this study. To some extent, this was truer for those who did not emigrate under force but made a significant thoughtful choice in the hopes of creating a meaningful life. More studies are needed on this topic.

During ageing itself, older adults may experience a change in their identity as their bodies change and they lose their roles and health.^40^ Our result implies that, for participants, leaving their country of origin behind has a dimension of loss of culture and leaving a piece of their identity behind. As migrants sought to both find and hold on to their identity, a sense of alienation was created and feelings of existential loneliness occurred.

Migrants’ identities were impacted by their life stories. Even in the early years in life, when the inclusion of an important collective dimension, affects how one’s identity is experienced,^41^ the course of one’s life itself also seems to be of importance. For example, ageing, with its inevitable bodily changes and losses in current life situation, has an impact on how one sees oneself. This part of life may, for some lead to an existential awakening with elements of existential loneliness. In our current study, participants often expressed that life-changing events such as migration affected their identity. In light of this, making room for older migrants to reconcile with their life story is of importance.

For older migrants, experience of existential loneliness may occur due to the absence of relationships, cultural customs and traditions from their country of origin as well as a feeling of not belonging. For older migrants, experience of existential loneliness may occur due to the absence of relationships, cultural customs and traditions from their country of origin as well as a feeling of not belonging. Structure and understanding the context in which the migrant is living in may help to cope with the feeling of otherness, that is, feeling of not belonging due to being different.^42–44^ Even though an individual may have lived in a new country for a long time, former structures such as language and culture can lead to existential loneliness. Relationships within the same social group seem to be needed for the migrants to not experience existential loneliness. A lack of belonging can indicate a lack of participation in the world^14^ and a lack of relationships with others can make individuals feel incomplete. Therefore, relationships with the same social group can fulfil the need to belong and may prevent migrants from experiencing existential loneliness. Communication is also key to taking part in ones’ experiences.^37^ The spoken as well as the cultural language can unite people and create a sense of meaning. However, when not speaking the same language there is a risk for alienation.^45^

Even though most of the participants in the current study had lived in Sweden for a long time, their cultural heritage played an important role in their experience of existential loneliness.

## Strengths and limitations

All interviews took place in Swedish by choice. This can be seen as both a strength and a limitation. The use of translation was discussed but due to the nature of the questions, the in-depth interview was performed without a third partner involved to avoid answers being filtered by an interpreter as well as to create an environment of trust for the participant. As the inclusion criteria included being able to communicate in Swedish, contextual factors needed to be considered when transferring the result. The participants themselves decided on their language abilities for the interview. Furthermore, three participants refused tape recording because they felt more secure if the interview were written down. The first author performing the interviews, listened most carefully, repeated the participants answers to avoid loss of data. The study was performed using a relatively small sample (n = 15) which therefore has implications for the trustworthiness of its findings. The participants represented in this study were mainly Europeans. To broaden and strengthen the result, it would be preferable to interview participants representing more continents. This limitation might reduce transferability; however, as there is, to our knowledge, not much written about the experience of existential loneliness among older migrants, we hope that this current study will contribute to understanding an aspect of existential loneliness.

In terms of participants’, there were only 3 men and 12 women which may be seen as a limitation. However, ages varied between age 66 and 99 years. By describing the procedure in analysis and using quotations from interviews, we hopefully enabled the reader to consider our interpretation to be valid and trustworthy as there always is a risk of subjectivity in interpretation of data, since this can be influenced by the interpreter’s life experience and ability.

## Conclusion and implications

This study indicates that migrants’ experience of existential loneliness derived from migration being a long-term process of significance. Regardless of whether older migrants’ made a thoughtful choice in emigrating or were forced to leave their countries due to difficult circumstances, their hope was to create a meaningful life. In old age, existential loneliness especially occurred as migrants’ sought reconciliation with life, reflected upon choices made in life and thoughts about death and dying.

Even though people from non-western countries will still constitute a minor part of the older population in the future, caring for older migrants’ may involve special challenges. Telling life stories was a way for participants become conscious of their prior experiences in life and how those experiences had affected them. When being able to put their life story in a broader context, earlier negative situations could help to bring about meaningfulness. Therefore, this study implies that participants life stories hold the key to handling existential loneliness. Older migrants’ life stories are most often complex and not always shared with others, but in telling their life stories, older migrants’ may find a way to maintain or find their identity in life situations felt to be meaningless. Meaningful relationships also seem to relieve the negative aspects of existential loneliness. As this research shows, being a migrant is a lifelong process and a factor for experiencing existential loneliness. In care for older adults, including older migrants, they are to be seen as unique individuals. This study also points out that cultural barriers can lead to a feeling of alienation and not belonging, which triggers experiencing existential loneliness.

To protect the older migrant’s autonomy and to facilitate the persons possibility to participate in the care is a prerequisite in order to provide good and ethical care. If an older migrant is not able to participate due to miscommunication or differences regarding cultural values, decisions related to the older migrant’s care will most often be left to healthcare professionals. In that case, there might be a risk that the health care professionals’ values will form the basis for decision concerning care of the patient. At best, the health care professionals’ decisions are consistent with the person’s, but this presupposes enough knowledge regarding the person and his or her life story, cultural understanding and creating mutual trust. In light of this, understanding ones’ existential loneliness can first be sustained through ethical considerations and when true partnership is shaped.

Besides knowledge about different aspects of ageing, health care professionals should establish a partnership were the older migrant’s life-story is at core in order to help older migrants understand and cope with existential loneliness. By highlighting the cultural diversities, the need for health professionals to show an openness, imply cultural competence and understanding existential loneliness among older migrants, comes to the fore. Once again, knowledge about older migrants’ life stories therefore may strengthen their own culture and self-perception. We emphasize the importance of ethical and cultural reflection on a regular basis in order for health care professionals to provide adequate care adapted for older migrants.
